# Pain characteristics and psychological factors that mediate the association between obesity and outcomes of interdisciplinary pain rehabilitation: a registry-based cohort study

**DOI:** 10.1080/07853890.2025.2517816

**Published:** 2025-06-18

**Authors:** Huan-Ji Dong, Agnes Genander, Elena Dragioti

**Affiliations:** a Pain and Rehabilitation Centre, and Department of Health, Medicine and Caring Sciences, Linköping University, Linköping, Sweden; b Department of Nursing, School of Health Sciences, Research Laboratory Psychology of Patients, Families and Health Professionals, University of Ioannina, Ioannina, Greece

**Keywords:** Chronic pain, obesity, depression, rehabilitation

## Abstract

**Background:**

Obesity is a common comorbidity with chronic pain and is closely related to functional and psychological complications of pain, which are also the main outcomes of interdisciplinary pain rehabilitation programmes (IPRP). How obesity influences IPRP outcomes is poorly understood. This study aims to investigate the role of pain characteristics and psychological factors before IPRP as mediators of the association between obesity and IPRP outcomes (i.e. pain intensity and psychological functioning).

**Methods:**

Sociodemographic variables, pain characteristics and psychological factors were retrieved from the Swedish Quality Registry for Pain Rehabilitation (SQRP). Data at baseline (pre-IPRP) and 1-year follow-up (FU-IPRP) were used in mediation analysis.

**Results:**

Of the 872 patients (mean age 45.8 ± 10.5 years, 80.3% female), 232 (26.6%) were obese (body mass index [BMI] ≥ 30 kg/m^2^). Patients with obesity reported higher pain intensity (*p* = 0.02), a higher number of pain locations (*p* < 0.001), and longer pain duration (*p* = 0.002) compared to non-obese patients. Significant improvements at FU-IPRP were found in pain intensity and psychological functioning for both obese and non-obese groups. Mediation analysis revealed that pain intensity, pain radiation and depressive symptoms at pre-IPRP reduced the improvement of pain intensity at FU-IPRP among the patients with obesity. Depressive symptoms and pain intensity (or pain radiation) also mediated changes in two psychometric outcomes of IPRP (dysfunctional scale and adaptive coper scale).

**Conclusion:**

At FU-IPRP, patients with obesity experienced improvements in pain and psychological well-being, which were mediated by pain intensity, pain radiation, and depression. The roles of these mediators need to be specifically addressed when designing a tailored IPRP for pain patients with comorbid obesity.

## KEY MESSAGES

- Patients with chronic pain and comorbid obesity had worse pain characteristics than non-obese peers before participating in a pain rehabilitation program.
- Pain intensity, pain radiation and depression mediated the improvements of rehabilitation for patients with chronic pain and comorbid obesity.

## 1. Introduction

Evidence suggests a link between obesity and chronic pain, although their causal relationships are not fully understood [1]. These relationships are assumed to be bidirectional and multifactorial. Several factors – e.g. inflammatory substances, depression, lifestyle behaviours (e.g. eating and activity level) and genetics – could contribute to both pain and obesity [1–3]. Recent systematic reviews reported adults with obesity have a higher prevalence of chronic pain and they also rated higher pain intensity [4–6]. The existing evidence suggests that obesity among patients with chronic pain is associated with greater physical disability and psychological distress [7]. Moreover, a high prevalence of depression and sleep problems in both pain and obesity is well documented [8–10]. Poor sleep can exacerbate pain and negatively affect the psychological state, which in turn can affect pain management and rehabilitation outcomes [11,12]. Obesity is often associated with poor sleep quality and sleep disorders, which could worsen pain experiences [9,13]. Sleep disturbance, measured by Insomnia Severity Index (ISI), might mediate the relationship between obesity and pain by exploring how sleep disturbances linked to obesity affect pain perception and rehabilitation outcomes. Overall psychological state plays an important role in the development of obesity-related musculoskeletal pain [14]. High pain catastrophizing, measured by Pain Catastrophizing Scale (PCS), is strongly associated with worse pain outcomes and obesity [14]. PCS scores could reveal how cognitive and emotional responses to pain contribute to the obesity–pain relationship and affect the efficacy of pain rehabilitation. Other examples, such as anxiety and depressive symptoms, are overrepresented in obese persons and influence both pain and obesity [15,16].

Coping is defined as the cognitive and behavioural strategies used by a patient to cope with the demands of a situation, including pain management [17]. To cope with chronic pain, patients often develop active or passive (i.e. emotional) strategies, which could be either adaptive or maladaptive [18,19]. For example, some pain patients adopt pain acceptance – an adaptive coping strategy – to keep their focus on functionality and their continued pursuit of valued activities and life goals despite pain. Pain acceptance is a core psychological component of Swedish interdisciplinary pain rehabilitation programmes (IPRP) [20]. IPRP is indicated for patients with complex chronic non-cancer pain conditions, which include a wide selection of pain-related consequences such as comorbidities, psychological distress, reduced work ability and sick-leave, perceived ill-health and low quality of life [21]. However, it is poorly understood whether IPRP reduces pain and pain-related psychological distress in chronic pain patients with comorbid obesity. Historically, studies have shown less adherence to medical plans by patients with obesity [22–24]. Newer studies show that this adherence is affected by stigma within the healthcare system, internalized stigma by the patient and healthcare plans that lack in their appropriateness and evidence [25,26]. Coping mechanisms are often developed in conjunction with social stigmas [27]. Since obesity is often accompanied by social stigma and reduced physical activity [28], it can lead to maladaptive coping strategies. Coping styles have been linked to pain outcomes, suggesting that how individuals cope with pain could influence the effects of obesity on pain management [29]. Multidimensional Pain Inventory (MPI) used in IPRP identifies the differences in coping profiles between patients with obesity and non-obesity, potentially mediating the effects of obesity on rehabilitation outcomes. Hence, the hypothesis could be that the MPI profiles mediate the relationship between obesity and the effectiveness of pain rehabilitation by reflecting poorer psychosocial functioning.

**Figure 1.**
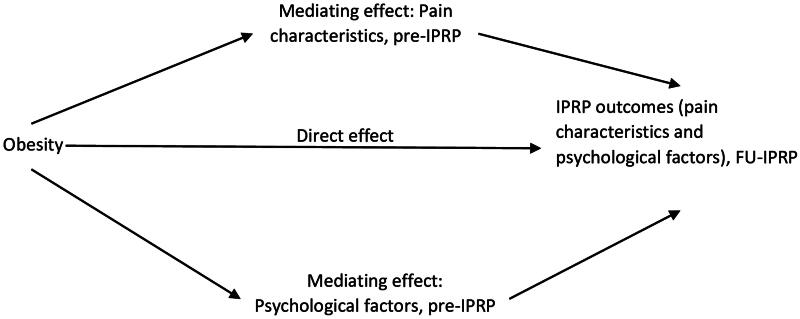
Conceptual diagram of a mediation model. In addition, we investigated the effects of moderating factors (age, gender, and education) upon the direct and mediating paths. The total effect is the sum of direct effect and indirect effects. IPRP: Interdisciplinary Pain Rehabilitation Program; Pre-IPRP: start of IPRP; FU-IPRP: one-year follow-up after IPRP.

It is unclear how pain characteristics and psychological factors, which mutually influence each other through obesity and chronic pain, influence IPRP outcomes. To fill this gap, using a cohort from the Swedish Quality Registry for Pain Rehabilitation (SQRP), we investigated the differences in these aspects between obese and non-obese patients with chronic pain. Our secondary aim was to understand if pain characteristics and psychological factors at baseline mediated the association between obesity and IPRP outcomes. Mediation analysis is particularly useful in exploring the indirect effects of an independent variable (i.e. obesity) on a dependent variable (i.e. one pain rehabilitation outcome) through one or more mediator variables (e.g. ISI, PCS, MPI, etc.). This analysis helps in quantifying the extent to which different factors contribute to or explain the relationship between obesity and pain, providing insights necessary for developing tailored interventions. Our previous study found patients with severe obesity (body mass index, [BMI] ≥ 35 kg/m^2^) experienced limited improvements after IPRP [30]. These findings elucidate a correlation that is not explained by evidence, thus mediators between comorbid obesity and pain with IPRP outcomes are investigated in this study. The pre-IPRP levels are worth being investigated to stop obesity stigma and negative perceptions [31]. We hypothesized that pre-IPRP pain and psychological aspects mediate the associations between obesity status and IPRP outcomes (Figure 1). If confirmed, this knowledge can be used to develop tailored IPRPs for pain patients with comorbid obesity.

The arrows in the figure (→) express a hypothesized relationship in agreement with prior studies, among the independent variable (obesity, to the left), the dependent variables (IPRP outcomes, to the right) and each mediator (pain characteristics and psychological factors at pre-IPRP, in the middle).

The direct effect, an arrow (→) from obesity to IPRP outcomes, indicates the effect of obesity on IPRP outcomes (FU-IPRP) after controlling for the mediator variables. An arrow (→) from obesity to pain characteristics (pre-IPRP) indicates an association between obesity and pain characteristics (e.g. pain duration or pain intensity). An arrow (→) from pain characteristics (pre- IPRP) to IPRP outcomes (FU-IPRP) indicates an association between the two variables (e.g. pain duration at pre-IPRP associated with pain intensity at FU-IPRP) when controlling for the effect of obesity.

The indirect effect is the path from obesity to IPRP outcomes at FU-IPRP *via* mediator variables (pain characteristics or psychological factors, pre-IPRP) by multiplying the standard coefficients for the two parts of the mediating path.

## 2. Methods

### 2.1. The Swedish quality registry for pain rehabilitation (SQRP)

The SQRP, initiated in 1998, is a tool for evaluation of the effects of IPRP in clinical practise (real world). Most Swedish specialized pain units (>90%) refer to data from this registry to assess patients and develop IPRPs. The data consist of Patient Reported Outcome Measures (PROM) on sociodemographic background and valid instruments measuring pain characteristics, function, and psychological profiles [32]. This Swedish national cohort collects data from 42 pain rehabilitation units in specialist care where the patients report higher rates of obesity, anxiety and depression compared with the primary care patient groups or non-tertiary care [32]. Three timepoints are used for data collection: first visit to the pain unit (pre-IPRP), immediately after IPRP (post-IPRP) and at the 1-year follow-up after IPRP (FU-IPRP). In this study, we analysed data measured at pre-IPRP and at FU-IPRP with a focus on pain characteristics and psychological factors.

### 2.2. Subjects

The study includes data from 872 patients with complex chronic non-malignant pain who participated in IPRP in Sweden in 2016. Patients were referred to evaluation for IPRP in specialist pain rehabilitation centres, where the most severe cases (complex pain conditions) were treated. IPRP is conducted in groups of six to eight patients, lasts between 6 and 8 weeks, and includes patient education, supervised physical exercise, training in simulated environments and cognitive behavioural therapy [33]. The interdisciplinary rehabilitation team includes a psychologist, physiotherapist, occupational therapist and a physician specialized in rehabilitation. The interdisciplinary team of professionals strives for a close collaborative approach with the patient and considers the patient’s specific goals. Each patient sets individual rehabilitation goals and makes an individual schedule in collaboration with the rehabilitation team. To be eligible for IPRP, the patients must take an active part in their behavioural changes and therefore show potential to change their behaviours. Before the initiation of IPRP, patients were generally assessed by the interdisciplinary rehabilitation team. The following inclusion criteria for the IPRP were used: (i) disabling chronic pain (on sick leave or experiencing major interference in daily life due to chronic pain); (ii) age between 18 and 65 years; (iii) no further medical investigations needed; (iv) written consent to participate and attend to the IPRP; (v) and agreement not to participate in other parallel treatments [34]. General exclusion criteria for participating in IPRP were severe psychiatric morbidity, addiction to alcohol and/or drugs, diseases that do not allow physical exercise and specific pain conditions recommended for surgical and/or medical interventions [34]. Pharmacological treatments were adjusted and optimized before IPRP. If needed, unimodal rehabilitation and non-pharmacological interventions were considered. The results were evaluated before referral to IPRP.

This registry-based cohort study used real-world data from the SQRP for specialist care (pain unit) in Sweden. The Ethical Review Board in Linköping, Sweden approved the study (Dnr: 2015/108-31). All participants were given written information about the study and gave their written consent. Personal data were processed according to the General Data Protection Regulation (GDPR) [35]. Reporting of the results was done in accordance with A guideline for Reporting Mediation Analyses (AGReMA) Statement (see Additional file 1) [36].

### 2.3. Measurement

#### 2.3.1. Sociodemographic variables

The sociodemographic variables retrieved from SQRP included age (years), gender (female/male), country of birth and educational level. Country of birth was categorized as Sweden, another Nordic country (i.e. Denmark, Finland, Iceland and Norway), another country in Europe, and any non-European country. Educational level was dichotomized into university or no university education. Working status was reported as unemployed, employed, self-employed or studying.

#### 2.3.2. Anthropometric variables

Self-reported height and body weight were collected in SQRP or completed at the first clinical evaluation at IPRP baseline. BMI was calculated as body weight (kg)/height (m)^2^. According to World Health Organization (WHO) criteria, BMI is divided into the following categories: underweight (<18.5 kg/m^2^), normal weight (18.5–24.9 kg/m^2^), overweight (25–29.9 kg/m^2^) and obese (≥30 kg/m^2^). Self-reported data can lead to overestimated height and underestimated weight. However, high correlations between measured weight- and height-calculated BMI and BMI derived from self-reported values have been reported (Pearson’s *r* = 0.89–0.97 for different age groups and gender) [37].

## 2.3.2. Pain characteristics

Pain intensity was determined using an 11-graded Numeric Rating Scale (NRS) ranging from 0 (no pain) to 10 (worst possible pain). Patients reported their experienced pain intensity during the previous 7 d (NRS-7d). Duration of pain (years) was determined by asking the question ‘when did you first feel the pain that you are currently experiencing?’. The number of anatomical regions with pain (i.e. pain extent) was measured [33]. Thirty-six predefined anatomical areas were used (18 on the front and 18 on the back of the body): head/face, neck/throat, shoulder, upper arm, elbow, forearm, hand, anterior aspect of the chest, lateral aspect of the chest, abdomen, sexual organs, thoracic aspect of the back, lumbar aspect of the back, hip/gluteal area, thigh, knee, lower leg and foot. The number of areas with pain was summed to form a score ranging from 0 to 36. The variable is denoted as Pain Region Index (PRI) and a high score indicates high spreading of pain (‘pain radiation’).

### 2.3.3. Psychological variables

The Hospital Anxiety and Depression Scale (HADS), which has 14 questions, is used to detect and measure the severity of anxiety and depression and to follow the symptoms over time. Each question has four alternative answers graded from 0 to 3 points. The answers are divided into two subscales, one for anxiety and one for depression, with 0–21 points. Each subscale has three levels, where a higher score indicates a more severe condition for anxiety/depression: 0–7 = no symptoms; 8‐10 = probably symptomatic; and 11–21 = severely symptomatic [38]. The total score of HADS was calculated by summing the scores within each subscale; a higher score indicates a high level of emotional distress [39]. Good internal consistency (Cronbach’s alpha of 0.89 at Pre-IPRP and 0.91 at FU-IPRP) was demonstrated in this study.

Multidimensional Pain Inventory – Swedish validated version [30] (MPI-S) assesses psychosocial and behavioural consequences of chronic pain [40]. MPI-S consists of 34 questions divided into two parts with a total of eight subscales. (The third part is omitted in the Swedish version). Each answer is graded from 0 to 6 points. Using established algorithms, we converted the results into three coping profiles: dysfunctional (MPI-DYS), interpersonally distressed (MPI-ID), and adaptive coper (MPI-AC). A value on 0–100 scale presents the degree to which patients match the three coping profiles [41,42]. A high score of MPI-DYS indicates high pain impact, affective distress, and severe functional limitations, whereas a high MPI-AC indicates low pain impact and high levels of functional activity. A high score of MPI-ID refers to one’s perceived poor social support and understanding from family members or significant others. Good internal consistency (Cronbach’s alpha of 0.80 at Pre-IPRP and 0.83 at FU-IPRP) was demonstrated in this study.

PCS measures the degree of catastrophizing thoughts related to pain. The scale consists of 13 questions with five alternative answers, graded from 0 to 4 points. Three subscales are obtained: PCS-rumination (four questions with a maximum score of 16), PCS- helplessness (six questions with a maximum score of 24) and PCS-magnification (three questions with a maximum score of 12) [29, 30]. Often, a total PCS score (maximum 52 points) is used; a higher score indicates more severe catastrophizing and a score over 30 is considered clinically significant [43,44]. Good internal consistency (Cronbach’s alpha of 0.91 at Pre-IPRP and 0.93 at FU-IPRP) was demonstrated in this study. ISI, a screening tool for insomnia, can be used to evaluate the severity of sleeping problems and to follow the effect of treatment. ISI includes seven questions about the perceived sleep of the patient in the previous two weeks. The answers range from 0 to 4 points and the total score from 0 to 28 points. A higher score indicates a more severe condition (0–7 = no clinical significance, 8–14 = small clinical significance, 15–21 = moderate insomnia, 22–28 = severe insomnia) [45]. Good internal consistency (Cronbach’s alpha of 0.88 at Pre-IPRP and 0.91 at FU-IPRP) was demonstrated in this study.

### 2.4. Statistical analysis

We performed data analysis using IBM SPSS statistics version 27 (IBM Corporation, Route 100 Somers, New York) and Jamovi version 2.3 (Sydney, Australia, retrieved from https://www.jamovi.org). The descriptive data were presented with mean and standard deviation (mean, ±SD), mean and 95% confidence interval (CI), or number and percentage (*n*, %). To determine the statistical difference between obese and non-obese groups, independent t-test or Mann–Whitney-U-test was used. Either paired samples t-test or Wilcoxon signed ranks was used to investigate differences within group between pre-IPRP and FU-IPRP. A *p* < 0.05 was considered significant. Moreover, effect sizes (ES) for within-group analysis were calculated using a calculator and reported as Cohen’s d (0.20–0.49 = small, 0.50–0.79 = moderate, >0.80 = large) for parametric variables and as Rank biserial correlation for non-parametric variables (0.10–0.29 = small, 0.30–0.49 = moderate, >0.50 = large) [46].

We then conducted mediation analysis to understand how much of the measured effect of the independent variable (obesity) on the dependent variable (IPRP outcomes, e.g. NRS-7d, MPI, HADS) was attributable to one or more potential mediator variables. Before conducting the mediation analysis, we selected the candidate variables in two steps. First, we included the variables that had significant changes from pre-IPRP to FU-IPRP within obese or non-obese groups. Second, we tested the correlations of these variables with obesity and IPRP outcomes. Variables not significantly correlated to obesity or IPRP outcomes were excluded from the mediation analyses [47]. Then, we examined the assumptions proposed by Baron and Kenny [48] and quantified the effects with both unstandardized (B) and standardized (β) regression coefficients. The following guideline was applied for evaluating ES: β: 0.01–0.08 = small effect; 0.09–0.24 = medium effect; and ≥0.25 = large effect [49]. Bias-corrected bootstrap CIs (BC-CI) were calculated using 5000 bootstrap samples to test indirect (i.e. mediating) effect of obesity on IPRP outcomes [50,51]. Last, we analysed the possible effects of three moderators: age, gender, and education. This hypothesis implies that the strength or even the direction of these relationships could vary based on these demographic characteristics. We focused on the direct effect of obesity on IPRP outcomes and on the important mediator paths. The effect is considered statistically significant when the 95% CI does not contain zero after bootstrapping.

## 3. Results

### 3.1. Sociodemographic variables

Sociodemographic characteristics for the participants are summarized in Table 1. At Pre-IPRP, the mean BMI for all participants was 27.5 (SD = 5.18), with 26.6% (232/872) classified as obese. Most participants were women (80.3%), and obese participants were slightly older than non-obese peers (47.1 ± 9.4 *vs.* 45.3 ± 10.9, *p* = 0.03). No other statistically significant difference was found between the two groups. A total of 215 previously unemployed participants (24.7%) returned to work or study at FU-IPRP, and this positive change was found to be similar in the obese group and non-obese group (23.6% *vs*. 27.7%, *p* = 0.184).

**Table 1.** Sociodemographic background, pain characteristics and psychological variables at pre-IPRP.

Variables	Total *N* = 872	Non-obese *n* = 640	Obese *n* = 232	*p* Value	Correlation with obesity
Female gender, *n* (%)	700 (80.3)	517 (73.4)	183 (81.7%)	0.535	–
Age, years	45.8 ± 10.5	45.3 ± 10.9	47.1 ± 9.4	0.033	0.07*
BMI					
Pre-IPRP	27.5 ± 5.2	25.1 ± 3.0	34.1 ± 4.1	–	–
FU-IPRP	27.3 ± 5.0	25.3 ± 3.2	33.5 ± 4.3	–	–
University education (*n* = 869), *n* (%)	244 (28.1)	187 (29.2)	58 (25.1)	0.241	
Country of birth, *n* (%)				0.602	–
Sweden	732 (84.2)	539 (83.4)	193 (86.5)		
Another Nordic country	15 (1.7)	13 (2.0)	2 (0.9)		
Another country in Europe	31 (3.6)	24 (3.7)	7 (3.1)		
Non-European country	91 (10.5)	70 (10.8)	21 (9.4)		
Working/Studying, *n* (%)					
Pre-IPRP	474 (54.4)	359 (55.4)	115 (51.3)	0.293	–
FU-IPRP	644 (73.9)	482 (74.4)	162 (72.3)	0.545	–
Pain characteristics					
PRI (*n* = 860)	15.1 (14.6–15.7)	14.4 (13.8–15)	17.2 (16.1–18.3)	<0.001	0.14***
Pain duration, years (*n* = 808)	9.1 (8.5–9.8)	8.5 (7.8–9.2)	10.9 (9.6–12.3)	0.002	0.10**
NRS-7d (*n* = 855)	6.6 ± 1.8	6.6 ± 1.8	6.9 ± 1.7	0.021	0.08*
Psychological variables					
MPI-DYS (*n* = 855)	38.5 (35.7–41.3)	36.6 (33.4–39.9)	43.5 (38.1–49.0)	0.025	0.07*
MPI-AC (*n* = 855)	31.1 (28.6–33.7)	32.3 (29.3–35.4)	27.8 (23.1–32.6)	0.202	−0.05
MPI-ID (*n* = 855)	30.3 (27.7–33.0)	31 (27.8–34.1)	28.6 (23.6–33.7)	0.589	−0.03
HADS-A (*n* = 866)	9.2 ± 4.5	9.2 ± 4.5	9.0 ± 4.7	0.457	0.02
HADS-D (*n* = 867)	8.7 ± 4.2	8.5 ± 4.2	9.2 ± 4.2	0.039	0.07*
HADS-total (*n* = 866)	17.9 ± 7.8	17.8 ± 7.8	18.3 ± 7.8	0.429	0.03
PCS, rumination (*n* = 832)	8.3 ± 4.1	8.3 ± 4.1	8.4 ± 4.1	0.705	0.01
PCS, helplessness (*n* = 837)	12.1 ± 5.3	12.0 ± 5.3	12.2 ± 5.4	0.685	0.01
PCS, magnification (*n* = 838)	4.5 (4.3–4.7)	4.5 (4.3–4.7)	4.7 (4.3–5.0)	0.45	0.03
PCS-total (*n* = 831)	24.9 ± 10.8	24.8 ± 10.8	25.2 ± 10.7	0.601	0.02
ISI sum (*n* = 836)	16.1 ± 6.6	16.0 ± 6.6	16.4 ± 6.5	0.48	0.03

Mean ± SD or mean (95% CI). IPRP: Interdisciplinary Pain Rehabilitation Program; Pre-IPRP: start of IPRP; FU-IPRP: 1-year follow-up after IPRP; PRI: Pain Region Index; NRS-7d: average pain intensity previous 7 d; MPI: Multidimensional Pain Inventory. DYS: dysfunctional profile; AC: adaptive coping profile; ID: interpersonal profile; HADS: Hospital Anxiety and Depression Scale; HADS-A: HADS-anxiety subscale; HADS-D: HADS-depression subscale; PCS: Pain Catastrophizing Scale; ISI: Insomnia Severity Index.

*p < 0.05, **p < 0.01, ***p < 0.001.

### 3.2. Pain characteristics at pre-IPRP and FU-IPRP

At pre-IPRP, some differences were found between the two groups (non-obese *vs.* obese, Table 1). Patients with obesity reported a higher number of pain locations (PRI, *p* < 0.001) and longer pain duration (*p* = 0.002) compared to non-obese patients. Patients with obesity also rated higher pain intensity (NRS-7d) than the non-obese patients at both pre-IPRP (small ES = 0.2, *p* = 0.02) and FU-IPRP (small ES = 0.27, *p* < 0.001). A reduction in NRS-7d at FU-IPRP was observed in both groups (moderate ES = 0.34–0.38, *p* < 0.01, Table 2).

**Table 2.** Effects of IPRP within groups of patients with obesity and non-obese patients at FU-IPRP, differences between groups and their correlations to obesity.

Variables	Non-obese (*n* = 640)	ES	Obese (*n* = 232)	ES	*p* Value (A *vs.* B)	Correlation to obesity
NRS-7d	5.6 ± 2.3	0.38**	6.2 ± 2.2	0.34**	<0.001	0.11**
MPI-DYS	24.3 (21.3–27.3)	0.44**	31.2 (25.8–36.6)	0.42**	<0.001	0.08*
MPI-AC	51.7 (48.1–55.2)	0.52**	43.6 (37.8–49.3)	0.48**	<0.001	−0.08*
MPI-ID	24 (20.9–27.0)	0.20**	25.2 (20.1–30.4)	0.13	0.705	0.02
HADS-A	7.5 ± 4.3	0.44**	7.6 ± 4.6	0.39**	0.516	0.01
HADS-D	6.8 ± 4.3	0.45**	7.1 ± 4.6	0.50**	0.029	0.04
HADS-total	14.2 ± 7.8	0.49**	14.7 ± 8.4	0.50**	0.427	0.03
PCS, rumination	6.3 ± 4.0	0.48**	6.5 ± 4.4	0.43**	0.502	0.02
PCS, helplessness	8.5 ± 5.5	0.65**	9.2 ± 5.6	0.62**	0.142	0.05
PCS, magnification	3.2 (2.9–3.4)	0.58**	3.2 (2.9–3.6)	0.56**	0.941	0.01
PCS-total	17.9 ± 11.0	0.68**	18.9 ± 11.6	0.65**	0.265	0.04
ISI	13.3 ± 7.1	0.43**	13.7 ± 7.3	0.40**	0.032	0.03

Mean ± SD or mean (95% CI). A *vs.* B: Non-obese group *vs.* Obese group. ES: effect size (FU-IPRP *vs.* pre-IPRP); NRS-7d: average pain intensity previous 7 d; MPI: Multidimensional Pain Inventory. DYS: dysfunctional profile; AC: adaptive coping profile; ID: interpersonal profile; HADS: Hospital Anxiety and Depression Scale; HADS-A: HADS-anxiety subscale; HADS-D: HADS-depression subscale; PCS: Pain Catastrophizing Scale; ISI: Insomnia Severity Index.

*p < 0.05, **p < 0.01.

### 3.3. Psychological variables at pre-IPRP and FU-IPRP

At pre-IPRP, statistical differences between the two groups (non-obese *vs.* obese) were only found in MPI-DYS and HADS-D (*p* < 0.05, Table 1). The FU-IPRP values are shown in Table 2 and significant differences between non-obese and patients with obesity were found for MPI-DYS, MPI-AC, HAD-D and ISI. Regarding effects of IPRP, ES varied from small to large and slightly higher in the non-obese group than the obese group. A large ES for MPI-AC improvement (ES = 0.52, *p* < 0.001) and a small ES for MPI-ID decrease (ES = 0.20, *p* < 0.01) were found in the non-obese group. For patients with obesity, a moderate ES for MPI-AC was found (ES = 0.48, *p* < 0.001).

### 3.4. Correlations and mediation analysis

Five variables at pre-IPRP (NRS-7d, PRI, pain duration, MPI-DYS and HADS-D) and three variables at FU-IPRP (NRS-7d, MPI-DYS and MPI-AC) showed significant correlations with obesity (Tables 1 and 2). The correlations between these variables are presented in Table 3.

**Table 3.** Pearson intercorrelations (correlation coefficients *r*) for the among the variables included in the mediation analyses.

	NRS-7d, pre-IPRP	PRI	Pain duration	HADS-D, pre-IPRP	MPI-DYS, pre-IPRP	NRS-7d, FU-IPRP	MPI-DYS, FU-IPRP	MPI-AC, FU-IPRP
NRS-7d, pre-IPRP	—							
PRI	0.24***	—						
Pain duration	0.04***	0.20***	—					
HADS-D, pre-IPRP	0.20***	0.12***	0.02	—				
MPI-DYS pre-IPRP	0.32**	0.08*	−0.14***	0.22***	—			
NRS-7d, FU-IPRP	0.46**	0.22***	0.07*	0.17***	0.18***	—		
MPI-DYS, FU-IPRP	0.27**	0.11**	−0.02	0.24***	0.46***	0.45***	—	
MPI-AC, FU-IPRP	−0.29***	−0.17***	−0.05	−0.39***	−0.19***	−0.52***	0.59***	—

PRI: Pain Region Index; NRS-7d: average pain intensity previous 7 d; MPI: Multidimensional Pain Inventory; DYS: dysfunctional profile; AC: adaptive coping profile; ID: interpersonal profile; HADS: Hospital Anxiety and Depression Scale; HADS-D: HADS-depression subscale.

*p < 0.05, **p < 0.01, ***p < 0.001.

**Figure 2.**
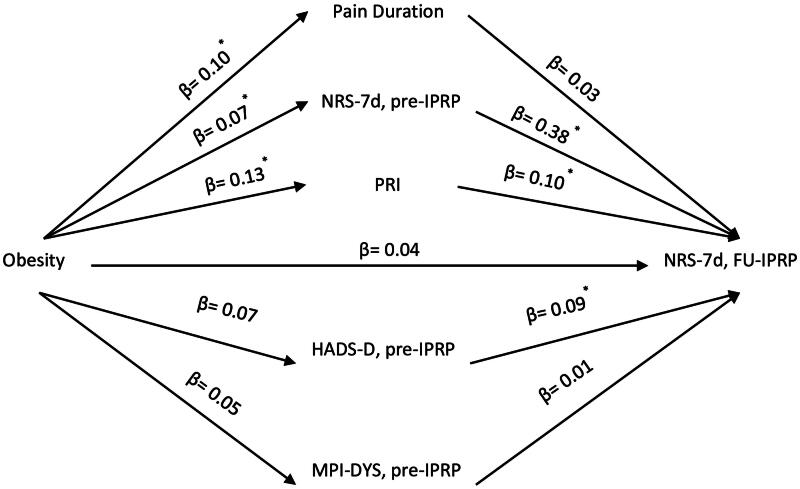
Conceptual diagram of the first mediation model. IPRP: Interdisciplinary Pain Rehabilitation Program; Pre-IPRP: start of IPRP; FU-IPRP: one-year follow-up after IPRP; NRS-7d: average pain intensity previous 7 d; PRI: Pain Region Index; MPI: Multidimensional Pain Inventory; DYS: dysfunctional profile; HADS: Hospital Anxiety and Depression Scale; HADS-D: HADS-depression subscale. *95% CI does not contain zero after bootstrapping.

**Figure 3.**
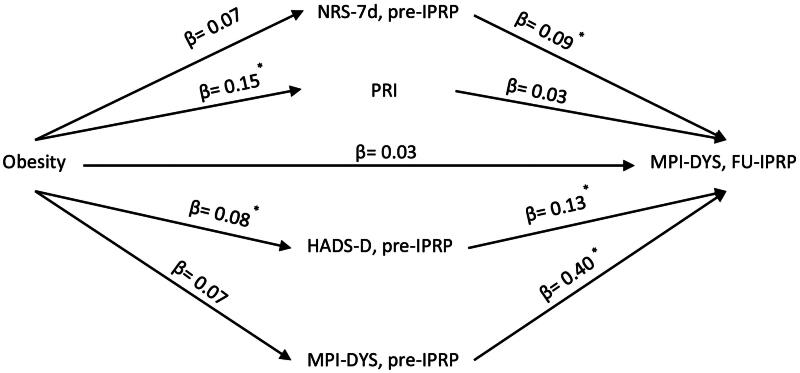
Conceptual diagram of the second mediation model. IPRP: Interdisciplinary Pain Rehabilitation Program; Pre-IPRP: start of IPRP; FU-IPRP: one-year follow-up after IPRP; NRS-7d: average pain intensity previous 7 d; PRI: Pain Region Index; MPI: Multidimensional Pain Inventory; DYS: dysfunctional profile; HADS, Hospital Anxiety and Depression Scale; HADS-D: HADS-depression subscale. * 95% CI does not contain zero after bootstrapping.

We performed three multiple parallel mediation analyses for the three IPRP outcomes: NRS-7d (Figure 1), MPI-DYS (Figure 2) and MPI-AC (Figure 3). Variables at Pre-IPRP were examined as mediators to explore their contributions to the associations between obesity and IPRP outcomes. No sociodemographic factor was significantly correlated with obesity and IPRP outcomes, so they were not included in the mediation analysis.

Path effects of the mediators on the association between obesity and NRS-7d at FU-IPRP are shown in Figure 2. The direct effect of obesity on NRS-7d at FU-IPRP was not significant (*B* = 0.21, 95% CI [−0.12, 0.5], *β* = 0.04), but there was a significant total effect (*B* = 0.46, 95% CI [0.1, 0.82], *β* = 0.09; medium ES) of obesity *via* the mediators. Specifically, we found significant indirect effects (mediation) of obesity through NRS-7d (*B* = 0.14, 95% CI [0.01, 0.28], *β* = 0.03; small ES), PRI (*B* = 0.06, 95%CI [0.02, 0.14], β = 0.01; small ES), and HADS-D (*B* = 0.03, 95% CI [0.002, 0.09], *β* = 0.01; small ES), although the path effect between obesity and HADS-D was not statistically significant. The full model with mediation and path estimates is reported in Table S1 in the supplementary material.

The second model regarding the association between obesity and MPI-DYS at FU-IPRP is shown in Figure 3. The direct path between obesity and MPI-DYS FU-IPRP was not statistically significant (*B* = 2.61, 95% CI [−2.54, 8.06], β = 0.03), but a significant total effect (*B* = 6.56, 95% CI [0.54, 12.57], *β* = 0.08; small ES) of obesity through all mediators indicates mediating effects. More analytically, we found significant indirect effects (mediators) of obesity on MPI-DYS through NRS-7d (*B* = 0.51, 95% CI [0.02, 1.41], *β* = 0.006; small ES) and HADS-D (*B* = 0.89, 95% CI [0.16, 2.09], *β* = 0.01; small ES), although not all their path estimates showed statistically significant results (Figure 3). The full model with mediation and path estimates is reported in Table S2 in the supplementary material.

**Figure 4.**
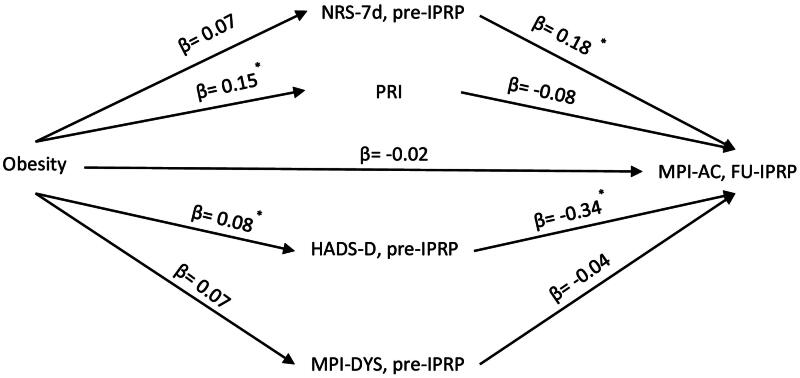
Conceptual diagram of the third mediation model. IPRP: Interdisciplinary Pain Rehabilitation Program; Pre-IPRP: start of IPRP; FU-IPRP: one-year follow-up after IPRP; NRS-7d: average pain intensity previous 7 d; PRI: Pain Region Index; MPI: Multidimensional Pain Inventory; DYS: dysfunctional profile; AC: adaptive coping profile; HADS: Hospital Anxiety and Depression Scale; HADS-D: HADS-depression subscale. *95% CI does not contain zero after bootstrapping.

The third model regarding the association between obesity and MPI-AC at FU-IPRP is shown in Figure 4. Like the other two models, the direct effect in this model was not significant (*B* = −2.31, 95% CI [−8.63, 3.78], *β* = −0.02). The total effect of obesity on MPI-AC was negative and significant (*B* = −7.51, 95% CI [−14.38, −0.64], *β* = −0.08; small ES) *via* the mediators. Two mediators – PRI (*B* = −1.17, 95% CI [−2.57, −0.29], *β* = −0.01; small ES) and HADS-D (*B* = −2.67, 95% CI [−5.17, −0.43], *β* = −0.03; small ES) – showed negative and significant indirect (mediating) effects on the association between obesity and MPI-AC at FU-IPRP, although the path estimate of PRI on MPI-AC was not statistically significant (Figure 4). The full model with mediation and path estimates is listed in Table S3 in the supplementary material.

#### 3.5. Moderating effects

**Figure 5.**
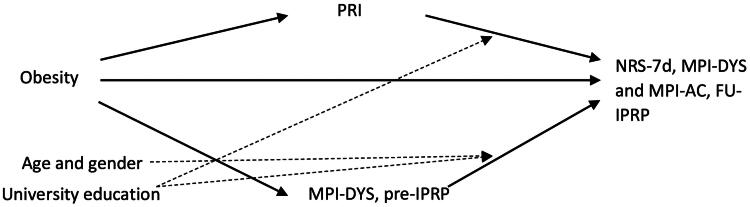
Moderating effects on the mediating paths. IPRP: Interdisciplinary Pain Rehabilitation Program; Pre-IPRP: start of IPRP; FU-IPRP: one-year follow-up after IPRP; PRI: Pain Region Index; MPI: Multidimensional Pain Inventory; DYS: dysfunctional profile; AC: adaptive coping profile.

None of the direct paths was significantly influenced by any of the potential moderators. The significant moderating effects on the mediating paths were shown in Figure 5. The age x MPI-DYS pre-IPRP interaction was statistically significant for MPI-DYS FU-IPRP (*B* = 0.007, 95% CI [0.002, 0.01]) and MPI-AC FU-IPRP (*B* = −0.009, 95% CI [−0.016, −0.002]). The gender x MPI-DYS pre-IPRP interaction was statistically significant for MPI-AC FU-IPRP (*B* = 0.25, 95% CI [0.07, 0.43]). Finally, the university education x MPI-DYS pre-IPRP interaction ((*B* = 0.009, 95% CI [0.001, 0.02]) and university education x PRI interaction (*B* = 0.06, 95% CI [0.02, 0.10]) showed statistically significant for NRS-7d FU-IPRP.

## 4. Discussion

Our results provide support for some pain characteristics and psychological factors as mediators of the relationship between obesity and IPRP outcomes. At pre-IPRP, patients with obesity had worse pain features than their non-obese peers regarding pain intensity, duration and pain radiation. Emotional distress (anxiety, depression and affective distress) showed weak correlations to obesity. Both patients with obesity and non-obese patients experienced improvements in psychological domains at FU-IPRP with varied ES. The mediation models indicated that severe pain features and depressive symptoms among patients with obesity affected their generally weaker outcomes of IPRP. Our study mirrors real-world data supporting that patients with obesity exhibit more severe pain characteristics and depressive symptoms than their non-obese peers. Given the bidirectional relationship between obesity and pain, future longitudinal studies are needed to explore whether these same factors have worsened obesity as a morbidity prior to the development of complex pain conditions.

Obesity, a common comorbidity of chronic pain, had a higher prevalence in this study population (26.6%) compared to the general adult population in Sweden (15%) [52]. This finding is in line with recent systematic reviews of prevalence studies [4,5]. It is very important to broaden the understanding of whether the comorbidity contributes to heavier burdens and worsens pain rehabilitation effects. Both obesity and chronic pain share many characteristics: they both are socially and often medically stigmatized; they both have complex causal mechanisms; they both require multidisciplinary treatments. Our findings support our hypothesis that patients with obesity had a more severe clinical presentation regarding pain aspects. Regarding the psychological burdens, a significantly higher score of HADS-D was found in the obese group than in the non-obese group. Translated to clinical practice, the groups differed by only one point in HADS-D within the same category of risk evaluation (0–7 = no symptoms; 8–10 = probably symptomatic; and 11–21 = severely symptomatic) at pre-IPRP. Importantly, at FU-IPRP, non-obese patients reduced their score from eight to six points, indicating a low risk for depression. Patients with obesity also had a more dysfunctional coping profile (MPI-DYS) at pre-IPRP than the non-obese group, which was correlated with higher pain intensity and depressive symptoms. Importantly, our findings indicate that patients who improved their adaptive coping (MPI-AC) at FU-IPRP demonstrated better rehabilitation outcomes, highlighting the role of coping strategies in the recovery process. While obesity alone was not significantly associated with psychological outcomes (MPI-DYS or MPI-AC) at FU-IPRP, the mediators explained the impact of obesity on rehabilitation results, reinforcing the importance of addressing these factors in IPRP. Further research could explore subgroup analyses to determine which patient profiles (e.g. those with high pain radiation and severe depression) most benefit most from IPRP, providing more targeted rehabilitation strategies.

We found both groups at FU-IPRP had significant improvements in several domains, but patients with obesity sustained improvements at a lower level than non-obese patients after IPRP. Interestingly, IPRP was still beneficial for both obese and non-obese participants with small improvements (e.g. pain characteristics, emotional distress and adaptive coping function) because IPRP is usually indicated for patients with complex chronic pain after all other available interventions are determined unhelpful with respect to pain alleviation. The significant differences in HADS-D scores likely reflect the more direct and robust links between obesity, depression, and chronic pain [1], whereas the absence of differences in PCS and ISI scores could be due to a combination of measurement sensitivities, sample characteristics and the multifactorial nature of these constructs. One previous study showed high levels of pain catastrophizing were more significant among osteoarthritis patients with BMI of 38 kg/m^2^ or greater than their peers with lower BMI (25–34 kg/m^2^) [53]. The evidence on the association between obesity and insomnia is inconclusive [54]. Further research might consider these variables to deepen understanding and refine intervention strategies.

Using mediation analysis, we provide further knowledge on how obesity might affect the IPRP outcomes. The high pain intensity and widespread pain suggest a severe burden for participants with obesity to improve *via* IPRP, which aligned with previous findings [4]. One of the three psychosocial profiles measured by MPI-scales (MPI-DYS) showed weak difference between the obese and non-obese groups at pre-IPRP. Obesity through pain features as well as depression disorder significantly affected the outcomes of two psychological profiles (MPI-DYS and MPI-AC) at FU-IPRP. Although not due to BMI status per se, the results suggest the mediating effect accounted for fewer improvements at FU-IPRP for the pain patients with obesity. In addition, we analysed the overall emotional distress by obtaining a HADS total scale [39], and the significant correlations to obesity and other IPRP outcomes disappeared. Therefore, we conclude that depression disorder played a more significant role than anxiety symptoms in the relationship between obesity and IPRP outcomes (i.e. decreasing pain intensity, decreasing dysfunctional scale and increasing adaptive coping function). This conclusion is in line with the current evidence that suggests a stronger significant association of obesity with depression than with anxiety disorders [16]. PRI as one of pain characteristics, may not have shown significant mediating effects possibly due to its more specific focus on the physical aspect of pain without encompassing the psychological or emotional dimensions that significantly impact rehabilitation outcomes. Its role as a mediator might be obscured by more dominant psychological factors like depression or broader measures of pain severity. The variability might also dilute the potential of PRI to consistently mediate between obesity and pain outcomes across a diverse patient population. While obesity can lead to mechanical stress and inflammation contributing to widespread pain [3,14], the specific number of affected regions might not capture the impact of obesity on pain experiences as effectively as other measures. In complex models where multiple variables potentially mediate the relationship between obesity and pain outcomes, the individual effect of a less direct mediator like PRI might be overshadowed by more potent mediators like depression.

When discussing obesity, weight stigma should be considered. It has been widely reported that patients with obesity are often reluctant to seek medical help for health issues due to weight stigma and bias [55,56]. Our findings encourage us to carefully consider how bias in medicine, social stigma, and patients’ internalized stigma can impact our clinical practice. Age, gender and education as moderators only affected the relationships between the clinical presentations (pain and psychological aspects at pre-IPRP) and IPRP outcomes, but not the indirect paths from obesity to pain or psychological aspects. The severe clinical presentations could be because patients with obesity delayed or avoided care, including participating in IPRP, and/or health professionals did not refer pain patients with comorbid obesity to specialized pain clinics earlier than non-obese peers when a pain condition presents. In Sweden, a referral from general practitioners or health providers from another specialty is mandatory for patients to visit a specialist whenever specialized health care is required. Since IPRP is a complex and costly intervention, several factors should be considered when making a clinical decision for IPRP participation. Our findings might be affected by some potential confounding factors not included in the mediation analysis (e.g. access to healthcare resources, socioeconomic status and additional comorbidities). From a clinical perspective, some possible explanations of this delayed care for pain patients with comorbid obesity are patient factors (health literacy, motivation, internalized stigma and self-confidence in reaching the rehabilitation’s goals), health provider factors (bias, knowledge of pain-related comorbidities and ability to deal with patients’ internalized stigma), and health system factors (resource prioritization for multidisciplinary care for comorbidities) [57–59]. To improve the management of chronic pain for obese pain patients, health care professionals should consider the perspectives of obese pain patients and investigate the attrition rate between referral and attendance in IPRP or other interventions.

It is evident that obesity interacts significantly with both pain and psychological factors, influencing pain rehabilitation outcomes. However, several small effect size of indirect effects on IPRP outcomes suggest that only aiming at weight loss might not lead to large improvements. The underlying mechanisms involved in the association of obesity and IPRP outcomes were more than BMI alone [60]. Given these findings, integrating obesity care into IPRP could be beneficial for patients with chronic pain and comorbid obesity [61]. For example, nutritional counselling should be tailored to individual characteristics and behaviours [2,62]. Individual rehabilitation plans to accommodate to the varying degrees of obesity and associated conditions [14]. This might include more gentle physical activity for those with severe pain in combination with tailored dietary interventions. Moreover, it may make a significant difference to provide additional psychological support to address potential barriers to lifestyle changes, such as motivation, self-efficacy and depression, which are extremely common in this patient group [29,63].

### 4.1. Strengths and limitations

Using a Swedish national cohort data in specialist care level, we reached relatively a large sample size (*n* = 872). Furthermore, we analysed the results from FU-IPRP to investigate the sustainable improvements after IPRP. The analysis was primarily cross-sectional in nature, with mediation models based on data collected at pre-IPRP and FU-IPRP. Mediation analysis was employed to explore potential indirect pathways – specifically how pain intensity, pain radiation and depression might mediate the relationship between obesity and IPRP outcomes. Although mediation models did not establish causality, they offered valuable insights into possible mechanisms underlying the observed associations. Future research could strengthen causal inferences by incorporating more frequent follow-up assessments or alternative study designs (e.g. randomized controlled trial, or control group with patients on the waiting list) that allow for a more robust evaluation of temporal relationships between obesity, psychological factors and pain rehabilitation outcomes.

This study has some limitations. First, we did not include any analysis of non-participants or drop-out cases because FU-IPRP only contains data from patients who completed IPRP. It remains unknown whether non-participants and drop-outs were more likely to be obese. Since patients with obesity experienced significant improvements in IPRP, it is important to note that high body weight is not a contraindication for participation. Second, it is worth noting the different BMI category classification for patients under 20 years old. In our study sample, eight of the participants were under 20 years old. According to WHO guidelines, overweight and obesity are defined as > +1SD and > +2SD, respectively [64]. Due to few participants under 20 years old, the results did not change when their BMI was recalculated according to WHO guidelines compared with previous categorization. Another aspect of BMI classification is about obesity class. We did not stratify participants by obesity class (e.g. BMI 30–34.9, BMI 35–39.9, BMI ≥ 40), which limited our ability to determine whether the observed mediation effects vary by obesity severity. This is particularly relevant given our previous study, which found that patients with severe obesity (BMI ≥35) had less benefit from IPRP. Future research should explore whether different obesity classes exhibit distinct mediation patterns to better understand the mechanisms underlying rehabilitation outcomes in this population. Third, significant values should be interpreted with caution due to generally small ES, and no correction was made for sensitivity analysis. However, bias is supposed to be considered in more than 10% missingness [65]. This was not the case in our sample population. On the other hand, from a clinical perspective, it is also important to recognize how the comorbid obesity and IPRP outcomes interact among the patients with complex pain conditions, although the effects could be small. Fourth, we focused on pain and psychological variables collected by instruments with relatively high response rates to conduct mediation analysis. We chose MPI as a core instrument to capture and integrate physical, psychosocial, and behavioural data for patients with chronic pain. The conclusion is only limited to the selected measures, and other aspects and IPRP outcomes (e.g. physical activity, interference or life control) were excluded. Future studies may also explore other outcome domains of IPRP since IPRP targets a whole person based on the biopsychosocial approach and goals in rehabilitation [21,66,67]. Finally, although the data for our analysis were rigorously sourced from the Swedish national cohort registry which is ensuring a high level of standardization and reliability, the prior registration of the research protocol is lacking. This was declared in the AGReMA checklist statement (see supplemental material).

### 5. Conclusion

At pre-IPRP, obese participants had higher pain intensity, greater pain radiation, longer pain duration, and higher scores regarding risk for depression than non-obese patients. Both obese and non-obese groups experienced improvements in several outcomes at FU-IPRP. At FU-IPRP, patients with obesity experienced improvements in pain and psychological well-being, which were mediated by pain intensity, pain radiation, and depression. The roles of these mediators need to be specifically addressed when designing tailored IPRPs for pain patients with comorbid obesity.

## Supplementary Material

Additional file 1.docx

Additional file 2.docx

## Data Availability

The datasets generated and/or analysed during this study are not publicly available as this was not a condition outlined to our participants but are available from the corresponding author on reasonable request.
