# Supplementary material for: Pain characteristics and psychological factors that mediate the association between obesity and outcomes of interdisciplinary pain rehabilitation: a registry-based cohort study
Source: Ann Med. 2025 Jun 18;57(1):2517816. doi: 10.1080/07853890.2025.2517816 (PMC12180319; doi:10.1080/07853890.2025.2517816)
Supplement: Additional file 2.docx [file IANN_A_2517816_SM1358.docx]

Supplementary material 2: Tables for mediation estimates and path estimates

Table S1: Mediation estimates and path estimates of obesity on pain intensity at FU-IPRP through pain and psychological factors

| Effect | B | SE | 95% CI Lower | 95% CI Upper | β | z |
| --- | --- | --- | --- | --- | --- | --- |
| Indirect effect of obesity on pain intensity at FU-IPRP via the mediators | | | | | | |
| Pain duration | 0.02 | 0.02 | -0.01 | 0.07 | 0.003 | 0.94 |
| Pain intensity, pre-IPRP | 0.14 | 0.07 | 0.01 | 0.28 | 0.03 | 2.03 |
| Pain spreading | 0.06 | 0.03 | 0.02 | 0.14 | 0.01 | 2.06 |
| HADS-D, pre-IPRP | 0.03 | 0.02 | 0.002 | 0.09 | 0.01 | 1.48 |
| MPI-DYS, pre-IPRP | 0.003 | 0.01 | -0.01 | 0.04 | 0.001 | 0.30 |
| Direct effect of obesity on pain intensity | | | | | | |
|  | 0.21 | 0.16 | -0.12 | 0.50 | 0.04 | 1.30 |
| Total effect of obesity on pain intensity (FU-IPRP) | | | | | | |
|  | 0.46 | 0.18 | 0.10 | 0.82 | 0.09 | 2.51 |
| Path estimates | | | | | | |
| Obesity ⇒ Pain duration | 2.06 | 0.82 | 0.44 | 3.72 | 0.10 | 2.52 |
| Pain duration ⇒ Pain intensity, FU-IPRP | 0.01 | 0.01 | -0.01 | 0.02 | 0.03 | 0.99 |
| Obesity ⇒ Pain intensity, pre-IPRP | 0.29 | 0.14 | 0.02 | 0.54 | 0.07 | 2.14 |
| Pain intensity, pre-IPRP ⇒ Pain intensity, FU-IPRP | 0.48 | 0.05 | 0.37 | 0.58 | 0.38 | 9.26 |
| Obesity⇒ Pain spreading | 2.34 | 0.67 | 1.01 | 3.72 | 0.13 | 3.51 |
| Pain spreading ⇒ Pain intensity, FU-IPRP | 0.03 | 0.01 | 0.01 | 0.04 | 0.10 | 2.86 |
| Obesity⇒ HADS-D, pre-IPRP | 0.70 | 0.35 | -0.003 | 1.35 | 0.07 | 1.96 |
| HADS-D, pre-IPRP ⇒ Pain intensity, FU-IPRP | 0.05 | 0.02 | 0.01 | 0.08 | 0.09 | 2.57 |
| Obesity⇒ MPI-DYS, pre-IPRP | 4.82 | 3.41 | -1.68 | 12.07 | 0.05 | 1.42 |
| MPI-DYS, pre-IPRP ⇒ Pain intensity, FU-IPRP | 0.001 | 0.002 | -0.003 | 0.005 | 0.01 | 0.38 |

Notes: Confidence intervals computed with method: Bias corrected bootstrap. Abbreviations: B, unstandardized beta; SE, standard error; CI, confidence interval; β, standardized beta.

Table S2: Mediation estimates and path estimates of obesity on MPI-DYS at FU-IPRP through pain and psychological factors

| Effect | B | SE | 95% CI Lower | | 95% CI Upper | | β | z |
| --- | --- | --- | --- | --- | --- | --- | --- | --- |
| Indirect effect of obesity on MPI-DYS at FU-IPRP via the mediators | | | | | | | | |
| Pain intensity | 0.51 | 0.34 | | 0.02 | 1.41 | 0.006 | | 1.48 |
| Pain spreading | 0.37 | 0.43 | | -0.41 | 1.34 | 0.004 | | 0.85 |
| HADS-D, pre-IPRP | 0.89 | 0.48 | | 0.16 | 2.09 | 0.01 | | 1.87 |
| MPI-DYS, pre-IPRP | 2.19 | 1.24 | | -0.13 | 4.69 | 0.03 | | 1.76 |
| Direct effect of obesity on MPI-DYS at FU-IPRP | | | | | | | | |
|  | 2.61 | 2.73 | | -2.54 | 8.06 | 0.03 | | 0.96 |
| Total effect of obesity on MPI-DYS at FU-IPRP | | | | | | | | |
|  | 6.56 | 3.07 | | 0.54 | 12.57 | 0.08 | | 2.14 |
| Path estimates |  |  | |  |  |  | |  |
| Obesity ⇒ Pain intensity, pre-IPRP | 0.26 | 0.14 | | -0.02 | 0.53 | 0.07 | | 0.85 |
| Pain intensity, pre-IPRP ⇒ MPI-DYS, FU-IPRP | 1.97 | 0.72 | | 0.58 | 3.41 | 0.09 | | 1.87 |
| Obesity ⇒ Pain spreading | 2.65 | 0.67 | | 1.34 | 3.99 | 0.15 | | 1.76 |
| Pain spreading ⇒ MPI-DYS, FU-IPRP | 0.14 | 0.15 | | -0.17 | 0.44 | 0.03 | | 1.87 |
| Obesity ⇒ HADS-D, pre-IPRP | 0.77 | 0.34 | | 0.10 | 1.43 | 0.08 | | 2.74 |
| HADS-D, pre-IPRP ⇒ MPI-DYS, FU-IPRP | 1.16 | 0.29 | | 0.59 | 1.72 | 0.13 | | 3.94 |
| Obesity ⇒ MPI-DYS, pre-IPRP | 6.17 | 3.42 | | -0.38 | 12.95 | 0.07 | | 0.90 |
| MPI-DYS, pre-IPRP ⇒ MPI-DYS, FU-IPRP | 0.35 | 0.03 | | 0.29 | 0.42 | 0.40 | | 10.42 |

Notes: Confidence intervals computed with method: Bias corrected bootstrap. Abbreviations: B, unstandardized beta; SE, standard error; CI, confidence interval; β, standardized beta.

Table S3: Mediation estimates and path estimates of obesity on MPI-AC at FU-IPRP through pain and psychological factors

| **Effect** | **B** | **SE** | **95% CI Lower** | | **95% CI Upper** | | **β** | **z** |
| --- | --- | --- | --- | --- | --- | --- | --- | --- |
| **Indirect effect of obesity on MPI-AC at FU-IPRP via the mediators** | | | | | | | | |
| Pain intensity | -1.12 | 0.64 | | -2.56 | 0.02 | -0.01 | | -1.74 |
| Pain spreading | -1.17 | 0.57 | | -2.57 | -0.29 | -0.01 | | -2.06 |
| HADS-D, pre-IPRP | -2.67 | 1.22 | | -5.17 | -0.43 | -0.03 | | -2.18 |
| MPI-DYS, pre-IPRP | -0.25 | 0.29 | | -1.14 | 0.10 | -0.003 | | -0.85 |
| **Direct effect of obesity on MPI-AC at FU-IPRP** | | | | | | | | |
|  | -2.31 | 3.18 | | -8.63 | 3.78 | -0.02 | | -0.73 |
| **Total effect of obesity on MPI-AC at FU-IPRP** | | | | | | | | |
|  | -7.51 | 3.51 | | -14.38 | -0.64 | -0.08 | | -2.14 |
| **Path estimates** |  |  | |  |  |  | |  |
| Obesity ⇒ Pain intensity, pre-IPRP | 0.26 | 0.14 | | -0.02 | 0.53 | 0.07 | | 1.86 |
| Pain intensity, pre-IPRP ⇒ MPI-AC, FU-IPRP | -4.36 | 0.86 | | -6.00 | -2.66 | -0.18 | | -5.07 |
| Obesity ⇒ Pain spreading | 2.65 | 0.67 | | 1.34 | 3.99 | 0.15 | | 3.96 |
| Pain spreading ⇒ MPI-AC, FU-IPRP | -0.44 | 0.17 | | -0.78 | -0.11 | -0.08 | | -2.54 |
| Obesity ⇒ HADS-D, pre-IPRP | 0.77 | 0.34 | | 0.10 | 1.43 | 0.08 | | 2.24 |
| HADS-D, pre-IPRP ⇒ MPI-AC, FU-IPRP | -3.47 | 0.34 | | -4.13 | -2.81 | -0.34 | | -10.35 |
| Obesity ⇒ MPI-DYS, pre-IPRP | 6.17 | 3.36 | | -0.24 | 12.95 | 0.07 | | 1.84 |
| MPI-DYS, pre-IPRP ⇒ MPI-AC, FU-IPRP | -0.04 | 0.04 | | -0.11 | 0.03 | -0.04 | | -1.11 |

Notes: Confidence intervals computed with method: Bias corrected bootstrap. Abbreviations: B, unstandardized beta; SE, standard error; CI, confidence interval; β, standardized beta.
